# Complementary and Alternative Therapies for Functional Gastrointestinal Diseases

**DOI:** 10.1155/2015/138645

**Published:** 2015-05-04

**Authors:** Jiande D. Z. Chen, Jieyun Yin, Toku Takahashi, Xiaohua Hou

**Affiliations:** ^1^Division of Gastroenterology and Hepatology, Department of Medicine, Johns Hopkins University School of Medicine, Baltimore, MD 21224, USA; ^2^Department of Surgery, Medical College of Wisconsin, Milwaukee, WI 53226, USA; ^3^Department of Medicine, Union Hospital, Huazhong University of Science and Technology, Wuhan 430030, China

Functional gastrointestinal diseases (FGID) are common in the world and account for more than 40% of clinical visits to gastroenterology clinics. Common FGID include gastroesophageal reflux disease (GERD), functional dysphagia, functional dyspepsia, gastroparesis, irritable bowel syndrome (IBS), functional constipation, diarrhea, and fecal incontinence. While pathogeneses of FGID are not completely understood, major pathophysiological factors include impaired gastrointestinal motility, visceral hypersensitivity, and psychological issues as well as disruption of the gut microbiota [1]. Gastrointestinal dysmotility is most common in FGID. For example, impaired lower esophageal sphincter function may lead to dysphagia in case of impaired relaxation during swallowing or GERD in case of reduced pressure or increased transient relaxation. In the stomach, reduced gastric relaxation during food intake may lead to impaired gastric accommodation, causing symptoms of early satiety and bloating; impaired antral peristalsis may lead to delayed gastric emptying, causing symptoms of nausea and vomiting. In the lower gut, impaired colon motility slows down transit, resulting in constipation, whereas a weak anal sphincter may lead to fecal incontinence. Visceral hypersensitivity is one of the major causes of pain and discomfort. It is commonly reported in patients with noncardiac chest pain, functional dyspepsia, and IBS. Depression and anxiety are commonly present in patients with FGID. Recently disruption of the gut microbiota has also been reported in patients with FGID.

Although FGID affect a large number of general populations, treatment options for FGID have been limited. Only a few medications have been developed for the treatment of FGID and few or none are available in the market currently depending on where one lives. Meanwhile, alternative and complementary medicine (CAM) has received more and more attention among patients with gastrointestinal diseases and gastroenterologists. In general population, the use of CAM was reported to range from 5% to 72% [2]. In patients with gastrointestinal diseases, the use of CAM was reported to be 40% in pediatric patients [3], 49.5% in patients with inflammatory dowel disease [4], and 50.9% in patients with IBS [5].

Major CAM methods that have been applied for the treatment of FGID include acupuncture/electroacupuncture, herbal medicine, and behavioral therapies. Electroacupuncture was initially designed to mimic manual acupuncture; electrical current was used to produce muscle contractions at the acupoint, mimicking the effect of manual manipulation of the needle inserted into the acupoint. Gradually, electroacupuncture has been evolved to function as neuromodulation or electrical nerve stimulation. That is, the parameters of electrical stimulation are chosen to alter certain functions of the body, such as release of certain hormones and/or neurotransmitter and alterations of certain physiological functions. Recently, a novel method of transcutaneous electroacupuncture (TEA) has been proposed: surface electrodes are used to replace acupuncture needles. This makes the therapy completely noninvasive and self-administrable. By replacing the acupuncture needles with cutaneous electrodes, the therapy can be administrated at home by patients and as frequently as needed. Acupuncture, electroacupuncture, and TEA have been shown to improve gastrointestinal intestinal motility and reduce visceral hypersensitivity in both humans and animal models of FGID [6]. A number of original research papers are included in this special issue. The study by X. Zhang et al. reported antiemetic effect of TEA in patients with chemotherapy and mechanisms involving serotonin and dopamine. The ameliorating effects of the noninvasive TEA on nausea and vomiting in the delayed phase are appealing as the common medical therapy has limited effects on nausea and vomiting in the delayed phase. The same TEA method was used in a study by F. Xu et al. The authors applied TEA in patients with functional dyspepsia and reported improvement in impaired gastric accommodation and gastric slow waves (electrical rhythms controlling peristalsis of the stomach). It was also reported that these effects were mediated via the vagal mechanisms. In another study by N. Da et al., electroacupuncture was used to treat patients with functional constipation and a comparison was made between shallow puncture and deep puncture. Both methods resulted in a significant increase in spontaneous bowel movement, and electroacupuncture with deep puncture was reported to be more potent than shallow puncture.

Herbal medicine has also been used for the treatment of FGID, such as STW 5 (Iberogast), Rikkunshito (also known as Liu-Jun-Zi-Tang), Daikenchuto, Simotang,* Taraxacum officinale*, modified Xiaoyao San, and Banxiaxiexin decoction [7]. In this special issue, Y. Saegusa et al. reviewed the treatment strategy of Rikkunshito for anorexia and gastrointestinal dysfunction. Rikkunshito was reported to improve gastric motility in both humans and animals and upper gastrointestinal symptoms such as dyspepsia, epigastric pain, and postprandial fullness in a number of clinical studies. Numata et al. in this issue reported improvement in functional constipation in poststroke patients with the use of Daikenchuto. A 4-week treatment with Daikenchuto significantly improved major symptoms or symptom scores associated with constipation in patients after stroke. In a placebo-controlled clinical study by Kamiya et al. in this special issue, Biobran, modified arabinoxylan rice bran, was reported to improve symptoms of diarrhea in IBS patients with diarrhea or mixed diarrhea and constipation, whereas no improvement was noted in the control group. It was speculated that the symptom improvement might be attributed to anti-inflammatory and/or immune modulatory effects of Biobran.

Behavioral therapies include cognitive behavioral therapy, hypnotherapy, relaxation exercise, mindfulness-based therapies, and biofeedback training. Most of these therapies have been applied for the treatment of FGID. One original study and one review paper are included in this special issue. In a study by Tang et al. an adaptive biofeedback training method was proposed and applied for the treatment of functional constipation due to paradoxical contractions of the rectum and the anal sphincter. In this method, the patients were trained to adequately control the contraction of the lower abdomen and relax the anal sphincter during straining; the actual manometric tracings showing the contractile activity of the rectum and anal sphincter were shown to the patients as visual feedbacks. A significant improvement in constipation-related symptoms was noted with both conventional and intensive biofeedback trainings.

In addition to original studies, this special issue also includes three reviews covering three major diseases of FGID, functional dyspepsia, IBS, and constipation. The paper by X. Wang and J. Yin provides a comprehensive and critical review on the applications of various CAM methods for the treatment of functional constipation. The review by M. Aucoin et al. provides a meta-analysis on the treatment of IBS using mindfulness-based therapies. The review by Y. Saegusa et al. presents a summary on the treatment of functional dyspepsia using a special herbal medicine, Rikkunshito.

*Jiande  D. Z.  Chen* *Jiande  D. Z.  Chen* *Jieyun  Yin* *Jieyun  Yin* *Toku  Takahashi* *Toku  Takahashi* *Xiaohua  Hou* *Xiaohua  Hou*
